# Yellow Fever Outbreaks in Unvaccinated Populations, Brazil, 2008–2009

**DOI:** 10.1371/journal.pntd.0002740

**Published:** 2014-03-13

**Authors:** Alessandro Pecego Martins Romano, Zouraide Guerra Antunes Costa, Daniel Garkauskas Ramos, Maria Auxiliadora Andrade, Valéria de Sá Jayme, Marco Antônio Barreto de Almeida, Kátia Campomar Vettorello, Melissa Mascheretti, Brendan Flannery

**Affiliations:** 1 Secretariat for Health Surveillance, Brazilian Ministry of Health, Brasilia, Brazil; 2 Department of Animal Science, Federal University of Goiás, Goiania, Brazil; 3 Health Surveillance Center, Rio Grande do Sul State Health Department, Porto Alegre, Brazil; 4 Epidemiological Surveillance Center, São Paulo State Health Department, São Paulo, Brazil; 5 Pan American Health Organization, Brasilia, Brazil; 6 Global Immunization Division, Center for Global Health, U.S. Centers for Disease Control and Prevention, Atlanta, Georgia, United States of America; Duke-NUS, Singapore

## Abstract

Due to the risk of severe vaccine-associated adverse events, yellow fever vaccination in Brazil is only recommended in areas considered at risk for disease. From September 2008 through June 2009, two outbreaks of yellow fever in previously unvaccinated populations resulted in 21 confirmed cases with 9 deaths (case-fatality, 43%) in the southern state of Rio Grande do Sul and 28 cases with 11 deaths (39%) in Sao Paulo state. Epizootic deaths of non-human primates were reported before and during the outbreak. Over 5.5 million doses of yellow fever vaccine were administered in the two most affected states. Vaccine-associated adverse events were associated with six deaths due to acute viscerotropic disease (0.8 deaths per million doses administered) and 45 cases of acute neurotropic disease (5.6 per million doses administered). Yellow fever vaccine recommendations were revised to include areas in Brazil previously not considered at risk for yellow fever.

## Author Summary

Yellow fever is a viral hemorrhagic disease transmitted by mosquitos, endemic in tropical regions of Africa and South America. Large urban outbreaks of yellow fever have been eliminated in the Americas, where most yellow fever cases result from human exposure to jungle or forested environments. Vaccination is effective but carries a risk of potentially fatal adverse events in a small number of vaccinees. In a large country such as Brazil, vaccination is recommended only in areas where there is a risk of exposure to yellow fever virus. We describe two outbreaks of yellow fever in areas without yellow fever vaccine recommendations. Numerous epizootics, or die-offs of non-human primates, were reported from areas with human cases. In response to the outbreaks and epizootic activity, over five million doses of vaccine were administered in previously unvaccinated populations, resulting in vaccine associated adverse events, six of which were fatal. The outbreaks resulted in expansion of areas with yellow fever vaccine recommendations, and highlight the need for safer yellow fever vaccines.

## Introduction

Yellow fever is an acute viral hemorrhagic disease transmitted by mosquitoes. Severity ranges from self-limited febrile illness to hemorrhagic syndrome with jaundice, multiple organ failure and death; severe cases are more likely to be detected and reported to passive surveillance systems [1], [2]. Yellow fever is considered endemic in tropical regions of Africa and South America. For endemic countries, the World Health Organization recommends vaccination of persons living in areas at-risk for yellow fever, as well as for epidemic control [3]. According to the 2010 revised yellow fever risk map, Brazil is one of 11 South American countries with endemic or transitional areas for yellow fever, and one of seven in which vaccine is recommended in only part of the territory [4].

In Brazil, yellow fever virus transmission is maintained in tropical forests in a sylvatic cycle first described in the 1930s [5], involving non-human primates and several species of tree-dwelling mosquitoes. Since several New World monkeys species develop fatal disease following yellow fever viral infection [2], sudden die-offs of non-human primates may signal yellow fever virus circulation and a potential exposure risk to humans. Surveillance for epizootic disease is recommended by the Pan American Health Organization [6], and is conducted in several Brazilian states as an early warning system for viral circulation to inform preventive vaccination [7]. In Brazil, sporadic human cases may also occur as a result of recreational or occupational exposures to jungle areas [8], [9].

Yellow fever vaccine developed from attenuated viral strains has been used in Brazil since 1939 [10]. Yellow fever vaccine recommendations must weigh the risk of exposure to yellow fever virus against the rare occurrence of fatal adverse events among vaccinated individuals [11], [12]. The Brazilian Ministry of Health recommends vaccination against yellow fever for persons who reside in or visit areas where transmission of yellow fever virus occurs [13]. However, yellow fever virus may also be transmitted from human to human by *Aedes* mosquitoes, resulting in urban epidemics. Although Brazil successfully controlled urban transmission in the 1940s through vector control and vaccination [14], re-establishment of *Aedes aegypti* in urban areas has resulted in recurrent epidemics of dengue fever and poses a risk for outbreaks of urban yellow fever [15]–[17]. Brazil's national yellow fever control strategy seeks to prevent human disease by identifying areas where the virus circulates, as well preventing re-introduction of urban epidemics through mass vaccination [11].

Since 1999, yellow fever has re-emerged in parts of Brazil that had been silent for several decades [9], [18]–[21], challenging prevention strategies and resulting in frequent revision of yellow fever vaccine recommendations [13]. Beginning in 2008, following yellow fever outbreaks in central and southeastern Brazil, northeastern Argentina and Paraguay [4], the Ministry of Health initiated enhanced surveillance for human and epizootic yellow fever viral activity during the usual seasonal period from October to June [8]. We describe two yellow fever outbreaks that occurred during the first year of enhanced yellow fever surveillance in Brazil, during the 2008–2009 season.

## Methods

### Ethics statement

This study involved analysis of routinely collected surveillance data and did not require ethical review according to the Brazilian National Committee for Ethics in Research. Personally identifiable information (patient name and information included on case report form) was available only to surveillance officers and was not used in this study.

### Surveillance methods and definitions

Yellow fever is a notifiable disease in Brazil. The national passive surveillance system of the Brazilian Ministry of Health receives reports of suspected cases of yellow fever and epizootic events from state and municipal health departments. Beginning in 2008, the Ministry of Health took several actions to increase sensitivity of surveillance and timeliness of response vaccination during the seasonal period of highest risk of yellow fever from October to June. Enhanced surveillance includes raising awareness of yellow fever among health workers, mandatory notification of persons with ictero-hemorrhagic syndromes, investigation of human deaths due to unknown causes, intensification of epizootic surveillance in non-human primates and immediate communication of investigation findings. The national yellow fever surveillance system coordinates epidemiological surveillance for human cases and epizootic events, as well as communication between health departments and public health laboratories and [13].

Suspected cases are defined as individuals presenting with fever accompanied by jaundice or hemorrhagic symptoms, who within the previous 15 days were exposed to areas considered at-risk of yellow fever or with evidence of yellow fever virus circulation. Suspected cases in persons who received their first yellow fever vaccination within 10 days prior to symptom onset are classified for surveillance as unvaccinated. Persons presenting with signs and symptoms of yellow fever (jaundice, abnormal laboratory values, hemorrhage or neurologic symptoms) who experienced symptom onset within 60 days of receipt of yellow fever vaccine identified through enhanced surveillance for ictero-hemorrhagic syndromes were investigated following a clinical and laboratory protocol as possible adverse reactions to vaccination [22]. Adverse events were classified as confirmed, probable, suspected, discarded or inconclusive, according to U.S. Centers for Disease Control and Prevention criteria [23]. Clinical, epidemiological and laboratory data, as well as vaccination history, are reviewed by expert committee that provides recommendation for final classification as previously described [22].

Laboratory confirmation for human cases includes the presence of yellow fever virus-specific antibodies detected by IgM-capture ELISA [24] or immunohistochemistry [25], [26], detection of yellow fever virus by reverse-transcriptase PCR [27] or isolation of yellow fever virus in cell culture [28]. Nucleotide sequencing of yellow fever virus is used to differentiate between wild-type viral infections and vaccine virus [27], [29].

An epizootic is defined as a sudden die-off of non-human primates in a small geographic area. Sightings of sick and dying monkeys or carcasses are reported to local health departments, which conduct investigations and collect specimens for laboratory testing. Epizootics of yellow fever are confirmed if: 1) yellow fever virus is detected by immunohistochemistry or reverse-transcriptase PCR or isolated from animal specimens [7], [20], [30], or 2) if multiple non-human primate deaths are clustered within a short time period or linked by similar hydrographic features or vegetation to neighboring areas with documented circulation of yellow fever virus.

A yellow fever outbreak is defined as two or more confirmed human cases with a common probable location of infection. In accordance with International Health Regulations, evidence of yellow fever virus circulation in an area in which the resident population has not been vaccinated against yellow fever is classified by the Brazilian Ministry of Health as a Public Health Emergency of National Importance, requiring immediate evaluation of the risk of viral dissemination and need for intervention [31], [32]. Recommendations for vaccination against yellow fever virus may be extended to “affected areas” (municipalities with confirmed yellow fever human cases or epizootics, or detection of yellow fever virus in mosquito vectors) and bordering municipalities. During yellow fever outbreaks, vaccination may be recommended beginning at 6 months of age; yellow fever is not recommended in infants younger than 6 months of age [3], [23].

### Statistical analysis

Surveillance data were tabulated in TabWin (version 3.2, Datasus, Brazilian Ministry of Health, Rio de Janeiro, Brazil) and maps were created in TerraView (version 3.2.1, INPE, São José dos Campos, Brazil). Analyses were conducted in EpiInfo (version 6.04d, Centers for Disease Control and Prevention, Atlanta, USA). Incidence of yellow fever was calculated as the number of confirmed cases divided by the total state population, stratified according to areas with or without yellow fever vaccination recommendations prior to the 2008–2009 outbreaks. Rates of adverse events were calculated per million doses administered in each state during the period.

## Results

### Yellow fever surveillance in humans

**Figure 1:**
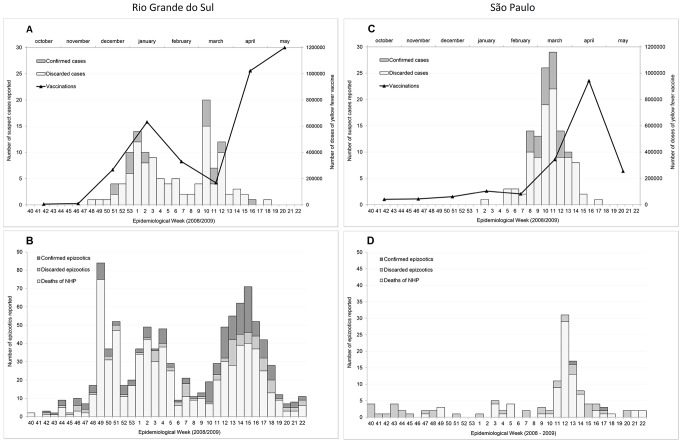
Notifications of suspected cases of human yellow fever and epizootics among non-human primates. Rio Grande do Sul state (Figures 1a and 1b) and São Paulo state (Figures 1c and 1d). Data from the national yellow fever surveillance system according to week of occurrence, October 2008–June 2009, and final classification of cases and epizootics: confirmed, discarded (laboratory negative) and unconfirmed (classified as death of non-human primate when no specimens were available for testing).

During the epidemic period from 28 September 2008 through 6 June 2009, 270 suspected yellow fever cases were reported to the Brazilian Ministry of Health. Of the suspected cases, 50 cases were classified as confirmed yellow fever based on laboratory criteria (n = 46) or epidemiologic linkage (n = 4). In the southernmost state of Rio Grande do Sul, there were a total of 21 confirmed cases (2.1 cases per million residents) with 9 deaths (case-fatality, 43%), including 3 cases (5.0 per million residents) in areas in which yellow fever vaccine was recommended and 18 cases (1.9 per million residents) in areas without vaccine recommendation prior to the outbreak (Figure 1a). In the most populous state of São Paulo, there were 28 confirmed cases (2.7 per million residents) with 11 deaths (case-fatality, 39%), all in areas without vaccine recommendations prior to the outbreak (Figure 1c). During the same period, only 1 (non-fatal) confirmed case (0.3 cases per million residents) resulting from sporadic exposure was reported from Mato Grosso state in central Brazil. Among other suspected cases, 196 (89.1%) were negative for antibodies to yellow fever and were classified as non-yellow fever cases and another 24 (10.9%) suspected cases had no epidemiological link with areas of transmission of yellow fever or had evidence of disease due to other causes that included dengue, leptospirosis, hantavirus infection and sepsis.

Among confirmed case patients, 35 (70%) were male; median age was 31 years (range, 3 days to 73 years). Characteristic symptoms of jaundice and hemorrhage were recorded on case report forms for only 17 (34%) and 18 (36%) confirmed cases, respectively, although transaminase levels were markedly elevated (Table 1). 37 (74%) were hospitalized for more than 24 hours and overall case-fatality was 40%. Age and gender distribution was similar among confirmed cases in the two most affected states.

**Table 1:** Patient characteristics, clinical findings and laboratory values for laboratory-confirmed yellow fever, Brazil, 2008–2009.

Variables	N = 50 (%)
Male gender	35 (70)
Age in years, median	31
Yellow fever vaccination*	1 (2)
**Clinical findings**	
Fever	36 (72)
Headache	27 (54)
Abdominal pain	20 (40)
Hemmorrhagic signs	18 (36)
Myalgia	18 (36)
Jaundice	17 (34)
Vomiting	15 (30)
**Laboratory values**	
Aspartate transaminase, mean mg/dl (range)	5132.2 (32–28900)
Alanine transaminase, mean mg/dl (range)	2480 (19–12600)
Total bilirubin, mean mg/dl (range)	4.6 (0–26)
Direct bilirubin, mean mg/dl (range)	2.7 (0.1–19.1)
Creatinine, mean mg/dl (range)	78 (13–280)
Urea, mean mg/dl (range)	4.2 (0.6–15)
**Yellow fever confirmation**	
IgM capture ELISA or immunohistochemistry	40 (80)
Viral isolation	1 (2)
Nucleic acid dectection	16 (32)
Histopathology	1 (2)
Epidemiologic linkage	4 (8)
**Evolution**	
Hospitalized >24 hours	37 (74)
Died	20 (40)

*1 confirmed case reported yellow fever vaccination nine years earlier; excludes 3 case patients with yellow fever vaccination 1–2 days before onset of symptoms.

One case patient with laboratory-confirmed yellow fever had received yellow fever vaccine 9 years earlier; none of the other case patients was considered effectively vaccinated. However, three individuals had received vaccine 1 to 2 days prior to experiencing symptom onset and were investigated as possible vaccine-associated adverse events; wild-type yellow fever infection was confirmed in two case patients by nucleotide sequencing while wild-type infection was considered most likely in the third case patient based on epidemiologic linkage to a laboratory-confirmed case. Four additional case patients received yellow fever vaccine after the onset of symptoms; identification of yellow fever virus confirmed wild-type infection in three case patients while the fourth case patient was classified as confirmed based on exposure to areas with yellow fever virus circulation prior to symptom onset.

**Figure 2:**
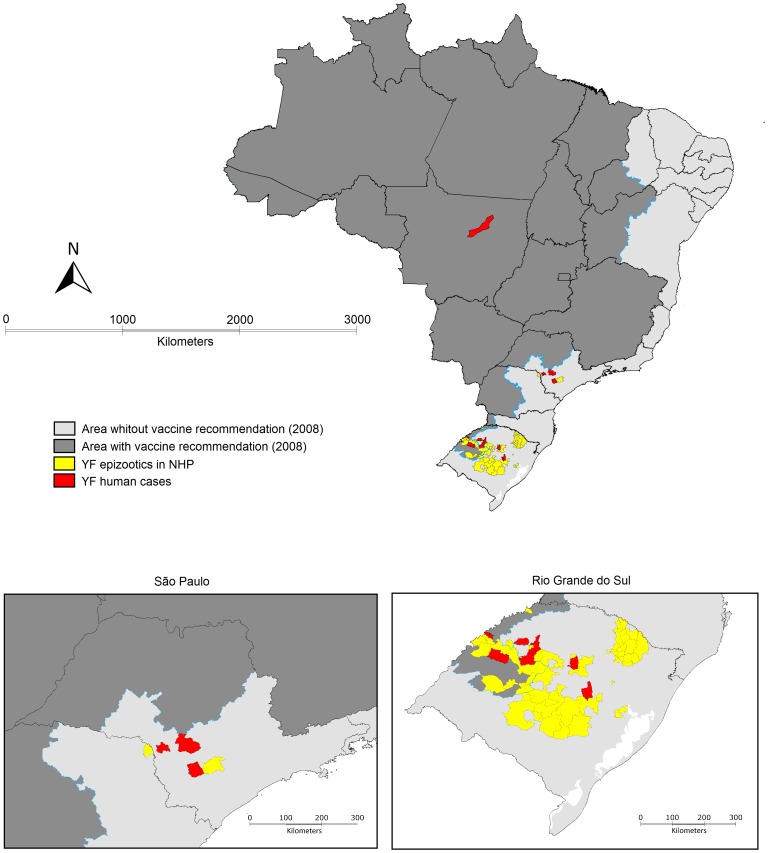
Location of confirmed epizootic events and human cases of yellow fever, Brazil, 2008–2009 (insets: states of São Paulo and Rio Grande do Sul).

A total of 46 (92%) confirmed cases occurred in areas in which vaccination against yellow fever had not been recommended prior to the 2008/2009 season (Figure 2). Probable locations of exposure for all confirmed cases were forested or rural areas, except for one case of perinatal exposure in an 8-day old infant [33]. The majority of cases were clustered in a small number of municipalities, including five in São Paulo state (Piraju [n = 11], Sarutaiá [n = 7], Buri [n = 5], Avaré [n = 4] and Tejupa [n = 1]) and nine in Rio Grande do Sul (Santa Cruz do Sul [n = 7], Vera Cruz [n = 4], St. Angelo [n = 3], Pirapó [n = 2], Bossoroca [n = 1], Espumoso [n = 1], Jóia [n = 1], Ijuí [n = 1], and Augusto Pestana [n = 1]). Of 14 municipalities in the two states identified as probable location of exposure of confirmed human cases, 12 were not previously considered at-risk for yellow fever and vaccination of residents had not been recommended prior to the epidemic. Yellow fever vaccination was recommended prior to the outbreak in two municipalities in northwestern Rio Grande do Sul following detection of yellow fever virus circulation in 2002 [30].

### Yellow fever surveillance in non-human primates

Epizootic activity involving deaths of non-human primates preceded human cases in both states that experienced yellow fever outbreaks (Figure 1). In Rio Grande do Sul, epizootic surveillance registered 950 reports of deaths among non-human primates during the period of enhanced surveillance; 947 [99%] involving howler monkeys of the genus *Alouatta*. Yellow fever virus circulation was confirmed in 173 (67%) of 259 events with samples available for testing, and 7 were confirmed by epidemiological linkage (Figure 1b). In Sao Paulo, 125 epizootics were reported and totaled 146 dead animals. Of these, 67 were of the genus *Alouatta* (45.9%), 56 *Callithrix* (38.3%), 14 *Cebus* (9.6%) and 9 were not identified (6.2%). Biological specimens were collected for testing from 64 (44%) of 146 animal carcasses, including tissue specimens from 58 animals and blood specimens from 23; the presence of yellow fever virus was detected in specimens from 2 separate epizootics (Figure 1d). Although 238 epizootic events were reported from other states, only 1 from Paraná state was confirmed as yellow fever.

In Rio Grande do Sul, epizootics were reported from all but one municipality identified as probable locations of exposure of confirmed human cases, and circulation of yellow fever virus among non-human primates was detected in the state 9 weeks before the occurrence of human cases (Figures 1a and 1b). In São Paulo, despite reports of epizootic activity throughout the epidemic period, yellow fever virus circulation among non-human primates was not confirmed until late March, 2009, following occurrence of human cases in late February 2009 (Figures 1c and 1d). In all, circulation of yellow fever virus was documented in 78 municipalities: 71 (91%) with confirmed epizootic activity (including 7 with both human and animal disease) and only 7 (9%) with confirmed human cases without confirmed epizootics.

### Vaccination

Prior to the outbreak in Rio Grande do Sul, yellow fever vaccination had been recommended in 59 municipalities in the northwest corner of the state (595,346 inhabitants); during and following the outbreak, vaccination was extended to 462 municipalities (93% of all municipalities in the state, with 9,963,267 residents), leaving only a small region along the Atlantic coast without vaccine recommendation. Following extension of yellow fever vaccine recommendations to affected areas, 3,636,722 doses of yellow fever vaccine were administered in Rio Grande do Sul (Figure 1a), reaching approximately 39% vaccination coverage in previously unvaccinated populations.

In São Paulo state, the number of municipalities with yellow fever vaccine recommendations increased from 332 (with 7,584,215 residents) to 452 (with 10,469,327 residents), covering 70% of the state and approaching metropolitan São Paulo. A total of 1,869,960 vaccine doses were administered in previously unvaccinated areas (Figure 1c), reaching 64% of the resident population.

In all, 5,506,682 million doses (69% of all doses administered in Brazil during the period) were administered in areas in which vaccination against yellow fever had not been recommended before the epidemic. In both states, numbers of doses administered peaked after the confirmation of human cases (Figure 1), although municipalities were included in the area with yellow fever vaccine recommendation at different times as the epidemic spread to new areas.

### Severe adverse events following yellow fever vaccination

During the same period, Brazil's national immunization program received 97 reports of severe adverse events among individuals who received yellow fever vaccine. Of these, 51 were classified by the national vaccine safety committee as associated with yellow-fever vaccine, including 6 cases of acute viscerotropic disease (incidence, 0.8 cases per million doses administered in Brazil), all of which were fatal, and 45 cases of acute neurologic disease (5.6 per million doses administered in Brazil) with no deaths. Of these, 40 cases (89%) were classified as aseptic meningitis; three cases (7%) were reported as encephalitis, one case was diagnosed as meningoencephalitis and one case as a right peripheral facial paralysis. However, two of the patients who had meningoencephalitis and encephalitis developed neurological sequelae that included difficulty walking and decreased visual acuity, respectively. Among the 6 confirmed cases of yellow fever vaccine-associated acute viscerotropic disease, median age was 31 years (range 4–44 years) and 2 (33%) case patients were male. Rates of vaccine-associated viscerotropic disease in the three states that reported cases ranged from 0.4 to 1.6 cases per million doses administered (Table 2). Among the 45 confirmed cases of yellow fever vaccine-associated acute neurologic disease, median age was 21 years (range 22 days - 66 years) with 26 (58%) cases among males. Rates of neurologic disease in the three states ranged from 0.8 per million doses administered in Santa Catarina to 11.0 per million in Rio Grande do Sul (Table 2). Two events were classified as vaccine-associated neurologic disease resulting from secondary transmission to breastfed infants [34], [35].

**Table 2:** Yellow-fever vaccine associated serious adverse events during outbreak response vaccination in Brazil, 2008–2009.

Syndrome	No. cases (no. deaths)	Male (%)	Median age, years (range)	Rate*	Median interval from vaccination to symptom onset, days (range)
State					
**Viscerotropic disease**	**6 (6)**	**2 (33)**	**31 (4–44)**	**0.8**	**3.5 (2–5)**
Rio Grande do Sul	2 (2)	1 (50)	39 (39)	0.5	4.5 (4–5)
São Paulo	3 (3)	1 (33)	30 (4–44)	1.6	3.0 (2–3)
Santa Catarina	1 (1)	0	23	0.4	4.0
**Neurologic disease**	**45 (0)**	**26 (58)**	**21 (0** ** **–66)**	**5.6**	**17 (2–40)**
Rio Grande do Sul	40 (0)	23 (58)	22 (0**–66)	11.0	19 (2–40)
São Paulo	3 (0)	2 (67)	24 (19–32)	1.6	9 (7–15)
Santa Catarina	2 (0)	1 (50)	4 (2–6)	0.8	17 (13–21)

* Cases per million yellow fever vaccine doses administered during outbreak response.

**Includes 22-day old infant with vaccine-associated neurologic disease following secondary transmission of yellow fever vaccine virus through breastfeeding [35].

## Discussion

These two yellow fever outbreaks in unvaccinated populations in the Brazilian states of Rio Grande do Sul and São Paulo during the 2008–2009 epidemic season challenged control strategies and resulted in revised vaccination guidelines for Brazil. Despite the relatively small number of confirmed cases and deaths, both outbreaks occurred in geographic areas without yellow fever vaccination recommendations, in which circulation of yellow fever virus had not been identified for four decades [9]. Although control vaccination was rapidly implemented following identification of human cases and epizootic events, the virus spread more quickly than expected and human cases continued to occur in newly affected areas. The experience in Rio Grande do Sul demonstrates the importance of active surveillance for yellow fever epizootics among non-human primates to inform vaccine recommendations [7]. Mass vaccination in previously unvaccinated populations may have prevented additional cases, but most vaccination occurred after the peak of the outbreaks. Yellow fever vaccine was associated with six deaths and multiple severe vaccine-related adverse events. Additional strategies are needed to prevent yellow fever outbreaks in unvaccinated populations until safer vaccines are available.

Mass vaccination has been associated with increased detection of adverse events since the first description of yellow fever vaccine-associated viscerotropic disease during intensified yellow fever vaccination in Brazil [22], [36], [37]. Vaccination of adults without prior immunity may increase rates of severe events, since risk is greatest with first vaccination and appears to increase with age [38], [39]. In addition, although detection rates of viscerotropic disease in Brazil are lower than estimated incidence based on United States surveillance data (0.4 per 100,000 doses administered) [39], improved surveillance during mass vaccination likely contributes to increased detection of adverse events [22]. The 2008–2009 outbreaks were the first in Brazil to identify rates of vaccine-associated neurotropic disease similar to those reported from adverse events surveillance in the United States (0.8 per 100,000 doses administered) [39], suggesting that enhanced surveillance and laboratory testing (specifically, real-time PCR for detection of yellow fever virus RNA in cerebrospinal fluid specimens) improved detection of neurologic events. Brazilian authorities have proposed universal childhood immunization against yellow fever to decrease risk of severe vaccine-associated adverse events later in life [11], based on the lower risk of viscerotropic disease in young children. However, due to uncertainty about the true risk, yellow fever vaccine is included in routine childhood immunizations only in Brazilian municipalities where vaccine is recommended for the entire population [40].

Factors associated with emergence of yellow fever are still poorly understood. It is unclear why yellow fever re-emerged in two non-endemic areas more than 1000 kilometers apart with limited evidence of viral circulation in other parts of Brazil. There had been widespread evidence of virus circulation in central Brazil during the preceding epidemic season (October 2007—June 2008), with confirmed human cases in 8 states and reports of epizootics in 14 states [8]. Circulation of yellow fever virus had been confirmed in northern São Paulo state and the western part of Paraná state (between Rio Grande do Sul and São Paulo) in early 2008. The role of climatic events (increased temperatures and high rainfall), high densities of susceptible non-human primate hosts and human exposure to mosquito vectors in forested areas may all have contributed [9]. Conditions may have favored viral spread to populations of susceptible non-human primate hosts during the interepidemic period, seeding outbreaks in the two previously unaffected areas. Better understanding of factors affecting viral spread and the dynamics of viral transmission would help to focus preventive immunization in unvaccinated populations.

**Figure 3:**
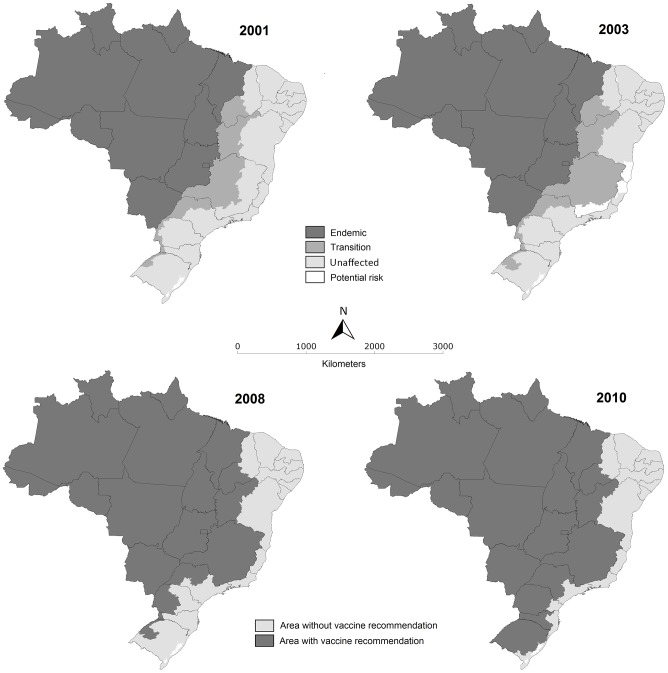
Evolution of geographic risk classification for yellow fever vaccination recommendations in Brazil, 2001–2010.

As a result of the 2008–2009 outbreaks, Brazil's yellow fever risk map was revised in 2010 to include large areas in which the population had not previously been vaccinated (Figure 3). This followed a change in 2008 by the Brazilian Ministry of Health to simplify classification of municipalities as those in which yellow fever vaccination is recommended or those without recommendation [13]. The 2008 revision harmonized state and federal recommendations, and the 2010 revision added vaccine recommendations in 334 municipalities in four states, with an estimated population of 8.6 million residents. In 2013, WHO revised yellow fever vaccine recommendations based on evidence that a single dose of YF vaccine confers life-long immunity against YF disease in most vaccine recipients, suggesting that booster doses may not be necessary [3]. However, cases of yellow fever have infrequently been documented in vaccinated individuals [3], as observed in one individual during the 2008–2009 outbreaks [13]. The Brazilian Ministry of Health continues to recommend re-vaccination against yellow fever every 10 years in areas considered at-risk for yellow fever, although priority is given to primary vaccination of previously unvaccinated persons in these areas. Vaccine recommendations may be revised as data become available from ongoing studies on the duration of immunity provided by yellow fever vaccine in adults and children.

During the past decade, from 2000 to 2010, the majority of human cases have been associated with exposures outside the Amazon River basin as yellow fever re-emerged in previously silent areas and unvaccinated individuals entered natural environments where yellow fever viruses circulate. As a result of changes in the epidemiology of yellow fever, the Brazilian Ministry of Health adopted new surveillance strategies, including enhanced monitoring of yellow fever virus activity during the epidemic season [13]. During the 2008–2009 epidemic season, enhanced surveillance contributed to improved laboratory diagnosis of suspected cases, detection of epizootic activity in affected areas and identification of vaccine-associated adverse events, especially neurologic disease. Earlier detection and treatment or more sensitive surveillance may be associated with lower case-fatality observed during 2008–2009 (40%) compared to the 2007–2008 season, with 49 confirmed cases and 28 (57%) deaths (Brazilian Ministry of Health, unpublished data). However, epizootic surveillance can only be effective in preventing human cases if there is adequate time to vaccinate or alert the population at-risk to avoid exposure of susceptible individuals. While active surveillance informed preventive vaccination in Rio Grande do Sul, epizootic events near São Paulo were not reported until after the outbreak had occurred. Additional strategies prioritized by the Ministry of Health included monitoring vaccination coverage in areas with yellow fever vaccine recommendations, syndromic surveillance for febrile icterohemorrhagic diseases for early case detection and surveillance for adverse events following yellow fever vaccination.

To prevent outbreaks as well as sporadic cases, public health authorities need to intensify efforts to ensure that individuals at highest risk of exposure are vaccinated. Unvaccinated individuals traveling to areas where vaccination is recommended should be vaccinated at least 10 days prior to travel. Public education is needed about the risk of disease and indications for vaccination, including contraindications and precautions for persons who might be at increased risk of severe adverse events. Efforts should also continue to develop a safer vaccine [41].

## Supporting Information

Checklist S1 **STROBE checklist.**
(PDF)
